# The Protective Effects of Flavonoids in Cataract Formation through the Activation of Nrf2 and the Inhibition of MMP-9

**DOI:** 10.3390/nu12123651

**Published:** 2020-11-27

**Authors:** Aaron Hilliard, Patricia Mendonca, Tanya D. Russell, Karam F. A. Soliman

**Affiliations:** 1 Division of Pharmaceutical Sciences, College of Pharmacy and Pharmaceutical Sciences, Florida A&M University, Tallahassee, FL 32307, USA; aaron.hilliard@famu.edu (A.H.); patricia.mendonca@famu.edu (P.M.); 2 Center for Advanced Professional Excellence, University of Colorado Anschutz Medical Campus, Aurora, CO 80045, USA; tanya.russell@cuanschutz.edu

**Keywords:** antioxidants, cataracts, MMP-9, Nrf2, oxidative stress, flavonoids

## Abstract

Cataracts account for over half of global blindness. Cataracts formations occur mainly due to aging and to the direct insults of oxidative stress and inflammation to the eye lens. The nuclear factor-erythroid-2-related factor 2 (Nrf2), a transcriptional factor for cell cytoprotection, is known as the master regulator of redox homeostasis. Nrf2 regulates nearly 600 genes involved in cellular protection against contributing factors of oxidative stress, including aging, disease, and inflammation. Nrf2 was reported to disrupt the oxidative stress that activates Nuclear factor-κB (NFκB) and proinflammatory cytokines. One of these cytokines is matrix metalloproteinase 9 (MMP-9), which participates in the decomposition of lens epithelial cells (LECs) extracellular matrix and has been correlated with cataract development. Thus, during inflammatory processes, MMP production may be attenuated by the Nrf2 pathway or by the Nrf2 inhibition of NFκB pathway activation. Moreover, plant-based polyphenols have garnered attention due to their presumed safety and efficacy, nutritional, and antioxidant effects. Polyphenol compounds can activate Nrf2 and inhibit MMP-9. Therefore, this review focuses on discussing Nrf2’s role in oxidative stress and cataract formation, epigenetic effect in Nrf2 activity, and the association between Nrf2 and MMP-9 in cataract development. Moreover, we describe the protective role of flavonoids in cataract formation, targeting Nrf2 activation and MMP-9 synthesis inhibition as potential molecular targets in preventing cataracts.

## 1. Introduction

Cataracts are the leading cause of blindness throughout the world. World Health Organization (WHO) estimates from 2010 indicate that cataracts are responsible for 51% of global blindness [1]. It is well known that aging is the main cause of opacification of the eye lens epithelia, either as a direct result of numerous environmental, nutritional, or metabolic injuries or as an indirect result of systemic or ocular diseases including diabetes, glaucoma, and retinal degenerative diseases [2,3].

As aging is an inevitable process, finding improvements in mechanisms to preserve sight from avoidable blindness is the focus of numerous research programs. Recent advances in non-surgical treatment options for cataracts, such as management of optimal refractive and glasses for glare reduction, can reduce the effect of cataract formation [4]. Cataract surgery is still a safe, well-validated treatment option, but not without caveats. Surgery-associated complications such as cystoid macular edema and posterior capsular opacification may arise and unavoidably cause irreversible blindness [5,6,7]. Rates from the US Cataract Patient Outcomes Research Team (PORT) indicate that cataract surgery has led to 3.21% for cystoid macular edema [5,6,7], 0.81% for posterior capsular opacification [5,6,7,8,9], and 1.1% for lens dislocation [5,6]. Thus, there is a need to identify non-surgical therapeutics with benefits outweighing the risks of surgery.

Oxidative stress can directly influence the solubility of the lens proteins, which increases the lens’s opacity. Oxidation seems to be a very early event that leads to cataracts formation [10,11]. The decline of antioxidants compounds levels may sign the changes that occur during senile cataract development [12]. With aging, the antioxidant potency is decreased, such as the diminished levels of glutathione or antioxidant enzymes expression [13]. Another vital contributor to cataract formation is the activity of matrix metalloproteinases (MMPs), which may decompose the extracellular matrix (ECM) of lens epithelial cells (LECs). MMPs were described to be associated with diabetic cataract [14,15,16], and increased levels of MMP-2 and MMP-9 in lenses stressed by oxidative stress, radiation, or transforming growth factor-β (TGF-β) were reported to contribute to cataract formation [17,18]. Numerous studies have shown evidence that alteration in the expression of MMPs may be associated with multiple cataract phenotypes [17], and thus, inhibition of their activity may have therapeutic potential.

It is crucial to identify novel compounds with antioxidant effects that could modulate molecular targets and prevent cataract formation. Additionally, compounds that can inhibit the expression of MMPs may help maintain the integrity of ECM and avoid further damages that could lead to cataracts. In this regard, plant-derived polyphenols, particularly flavonoids, have garnered attention due to their presumed safety and efficacy, nutritional, and antioxidant therapeutic effects. These compounds have been described to activate the nuclear factor (erythroid-derived 2)-like 2 (Nrf2), a transcription factor involved in the regulation of cytoprotective genes and cellular protection against oxidative stress contributing factors such as aging, disease, and inflammation. Flavonoids also have shown an inhibitory activity in the expression of MMPs in ocular cells, such as human LECs. Therefore, this review describes (1) the association of oxidative stress in cataract formation, (2) the role of the transcription factor Nrf2 in reducing oxidative stress and its protective effect against cataract formation, (3) the role of epigenetic in Nrf2 expression, (4) MMP-9 expression in cataract formation, and (5) how MMP inhibitors may be useful tools for cataract prevention. Moreover, we discuss the use of flavonoid supplements that may increase Nrf2 activity and attenuate MMP-9 expression, which may be new targets to prevent or slow the lens cataract progression.

## 2. Types and Causes of Cataract Development

The cataracts types are classified according to their location on the lens. Nuclear cataract, usually a result of advanced age, is the one found at the center of the lens; cortical cataract is usually related to diabetes and corresponds to the one that extends from the outside to the center of the lens; and subcapsular cataract which is associated to radiation from microwave, diabetes, and patients who take steroids and develops at the lens back portion [19].

A cataract is considered a multifactorial disease and can develop from several reasons such as congenital defects, age, injuries, systemic inflammation and degeneration, endocrine disorders and biochemical abnormalities, drug abuse, radiation, and oxidative stress [20]. Hereditary genetic anomalies correspond to one-third of the factors that contribute to congenital cataracts [21], and it may happen in the presence or not of aniridia, microphthalmia, developmental anomalies in the anterior chamber, degeneration of the retina, or other genetic disorders such as chromosome abnormalities [22]. A congenital cataract may also be caused by malnutrition or infection during pregnancy [23], as well as endocrine disturbance [22], drug abuse, or radiation exposure [23]. A higher incidence of cataracts was observed in women compared to men, where African and Hispanic Americans seem to have twice the risk compared to Caucasians [21]. A senile cataract occurs in people over 50, and it becomes more often and severe in the elderly [24]. Aging is the most frequent risk factor for cataracts, and it is linked to decreased levels of glutathione and broad modification of nuclear proteins, including change in color and oxidation [19].

There is a higher chance of cataract in smokers, linked to increased harshness of nuclear opacities. Inhaled smoke contains aromatic compounds that modify lenticular components oxidatively [25]. Traumatic cataracts can be developed after eye injuries such as physical injuries and eye lens capsule discontinuation. With the capsule breaking of the outer lens, a water swelling of the inner lens occur and becomes white, leading to lens proteins denaturation. Complicated cataract refers to a type that is not a primary disease to the eye but comes from other diseases, such as some of the drugs used in glaucoma treatment [21], eye inflammation, and uveitis originated from some autoimmune diseases [23]. A metabolic cataract is caused by endocrine disorders such as diabetes mellitus, which is connected with the development of several systemic and ocular complications that may lead to vision loss [26,27]. In vivo and in vitro studies have indicated that uncontrolled diabetes may lead to hyperglycemia, which is linked to ocular tissues with non-enzymatic protein glycation [28], osmotic stress [29], and oxidative stress [30], leading to cataracts. Many drugs can also contribute to cataracts, including steroids [31] and neuroleptic drugs employed in psychiatric disorders treatment [32]. Long-acting cholinesterase inhibitors might stimulate the anterior sub-capsular granular type of reversible cataract [23]. Several toxins, including synthetic medications, were reported to trigger cataracts, including acetone, dinitrophenol, cresol, and paradichlorobenzene. Alcohol consumption elevates the possibility of nuclear, cortical, and posterior sub-capsular cataracts since the lenses are susceptible to oxidative stress and the direct toxic effect of alcohol [33,34].

## 3. Pathophysiology of Cataracts

The human eye lens epithelium comprises the middle layer of the lens and contains a monolayer of metabolically active (i.e., oxidation) epithelial cells [35]. During aging, the migration of LECs to the inner fibrous portion of the lens occurs and it causes them to become lens fibers, gradually compressing and forming nuclear opacity [35]. Additionally, oxidative stress may induce degradation and aggregation of the lens α, β, and γ crystalline proteins, which comprise 90% of lens proteins, resulting in opacity and cataract formation [4,35,36,37,38]. Other major contributors to oxidative stress in the lens include damage in DNA, lipid peroxidation, and an imbalance in calcium homeostasis [39]. Glutathione, one of the main antioxidants in the eye lens, typically protects lens proteins against reactive oxygen species (ROS) including hydroxyl radicals, superoxide, and hydrogen peroxide (H_2_O_2_) in healthy lenses; reduced glutathione converts to its oxidized form when it reacts with ROS and is reinstated through glutathione reductase action, which is synthesized and regenerated on the lens cortex [40]. H_2_O_2,_ a significant contributor to oxidative stress and the pathogenesis of cataracts, is generally removed by glutathione or by the activity of catalase and glutathione peroxidase [41]. Conversely, a decrease in these protective mechanisms’ activity occurs with aging, resulting in elevated H_2_O_2_ levels in the lens, inhibition of membrane lipids and proteins that work as transporters (Na+K+ATPase), and ultimately leading to lens epithelial cell death and opacity [1,42].

Moreover, oxidative stress has been described as a crucial factor in cataract genesis. It rises as the human lens age, leading to a significant increase in protein concentration in cataractous lenses [37]. The imbalance between ROS production and the cellular antioxidant defense system originates from the oxidative stress process. In the eyes, cells ROS may induce toxic biochemical reactions, including membrane lipids peroxidation and protein injure, leading to intracellular protein aggregation and precipitation [37]. The ocular lens is at constant risk of photooxidative injure because of ambient oxygen and exposition to light, leading to cataract. The oxygen-free radicals can affect the lens crystallins, which form the opacities, and proteolytic enzymes that eliminate damaged proteins. Therefore, the upregulations of antioxidants including glutathione, catalase, and superoxide dismutase may reduce the modifications during senile cataracts development [43].

## 4. Nrf2 Reduces Oxidative Stress and Inflammation Levels

**Figure 1:**
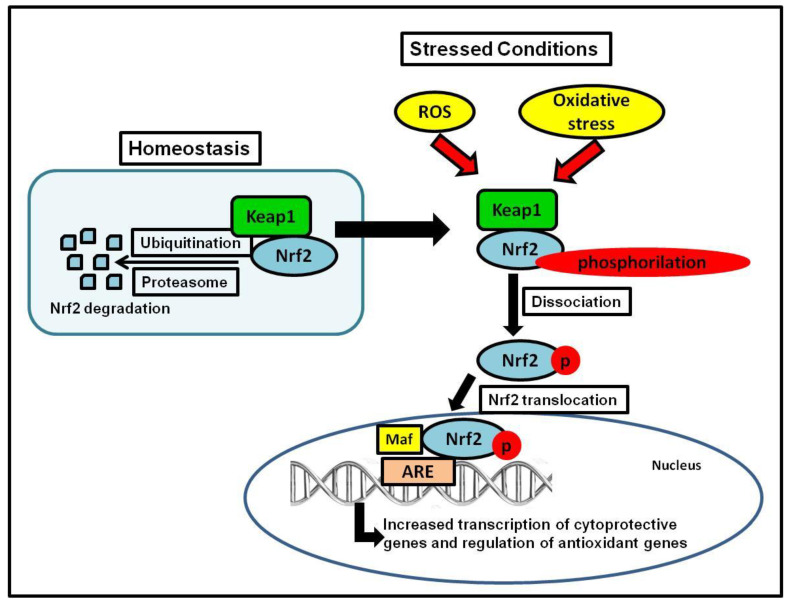
Keap1-Nrf2 system. The figure shows Nrf2 activation during stress conditions, which leads to the transcription of cytoprotective and antioxidant genes.

Nrf2 controls the redox homeostatic gene regulatory system, and the Nrf2 Kelch-like ECH-associated protein 1 (Keap1) complex is known as one of the most important mechanism in the cellular defense against oxidative stress [35,36]. Nrf2 regulates approximately 600 cytoprotective genes [36], serving as a critical nuclear transcriptional inducer by binding to the antioxidant response element (ARE) in DNA promoters and controlling the transcription of many antioxidant genes such as glutathione reductase, thioredoxin, and glutathione-S transferase [44,45]. To maintain homeostasis as a sensor of oxidative stress, Keap1 serves as the main inhibitor of Nrf2, and regularly targets Nrf2 for ubiquitination and subsequent degradation of 26S proteasomal to keep Nrf2 basal levels [46]. During unstressed conditions, Nrf2 is kept in the cytoplasm bound to Keap1 in a relatively rapid interaction; with a short half-life of 13–21 min, this rapid turnover ensures low, basal levels of Nrf2 [36,47,48,49]. During stressed conditions (i.e., oxidative or endoplasmic reticulum (ER) stress), Nrf2 separates from Keap1, it is phosphorylated, translocated into the nucleus, and it stimulates the transcription of antioxidant genes controlled by ARE, ultimately initiating the detoxification of ROS by the regulation of glutathione levels [50] (Figure 1).

The discrepancy between ROS production and the antioxidants’ capacity to detoxify the reactive intermediates can lead to oxidative stress [3,51]. Oxidative stress may be associated with several abnormalities leading to cell apoptosis and death [52,53]. ROS are essentially short-lived and extremely reactive and are stimulated by a diversity of intracellular pathways, including by-products of normal aerobic metabolism or messengers in various signaling pathways [3]. As mentioned, enhanced oxidative stress and the decrease of antioxidant defense are thought to be two main contributors to the pathogenesis of age-related cataract development. Many studies have probed into the molecular details of oxidative stress involvement. In particular, mechanisms of oxidative stress has been implicated in the activation of transcription factors such as Nrf2 and Kelch-like erythroid cell-derived protein with CNC homology (ECH)-associated protein 1 (Keap1), both involved in the activation of cell survival and death mechanisms [35,54,55] Phase II antioxidants such as heme oxygenase 1 (HO-1) are regulated by the Nrf2. Overproduction of ROS leads to the suppression of Nrf2-dependent antioxidant protection in LECs [42,51].

Previous studies have revealed that the transcription factor Nrf2 controls the expression of phase II detoxifying enzymes and antioxidant genes that play a role in the cell defense against various injuries through their anti-inflammatory effects, hence modulating the disease course [56,57,58]. High levels of oxidative stress and elevated production of the ROS may help the formation of lipid peroxides that contribute to aging pathologies that have a role in systemic and retinal degenerative diseases, including diabetes and inflammation. These diseases are considered significant risk factors for the formation of cataract [59].

## 5. Nrf2 Activation and Cataracts

Numerous studies have examined the antioxidant and anti-inflammatory effects of several Nrf2 inducers that may serve as potential anti-cataract therapeutic compounds. Nrf2 protective effects may be increased by pharmacologic or molecular modulations, where increased antioxidant and anti-inflammatory effects can offer new and helpful targets for devastating diseases that lead to blinding [60]. Acetyl ester of the trimethylated amino acid L-carnitine (ALCAR) has been shown to prevent cataract formation in rat models by increasing the levels of antioxidant proteins controlled by Nrf2 and decreasing proteins induced by ER stress in homocysteine-treated cells [61]. Morin (3, 5, 7, 20, 40-pentahydroxyflavone), widely used in herbal medicines, has been shown to increase the Nrf2 protein levels and stimulate the extracellular signal-regulated kinase (ERK)-Nrf2 signaling pathway in human LECs, leading to the upregulation of HO-1 and Nrf2 cytoprotective effects against oxidative stress [62]. Plant-extracted isothiocyanate 1-isothiocyanato-4-methyl-sulfinyl butane (SFN) has gained attention as a potential nutritional anti-cataract therapy by its ability to increase the activity of thioredoxin reductase in the lens of mouse, which prevents oxidative stress and cataract formation when consumed [63]. The multi-target neuroprotective drug, DL-3-n-butylphthalide (NBP), is widely utilized to treat ischemic stroke patients and diminishes oxidative damage, enhances the function of the mitochondria, lessens inflammation, and decreases neuronal apoptosis [64]. NBP has also been shown to induce the expression of Nrf2 in the lenses of diabetic rats [64] and maybe a promising anti-cataract therapeutic option with further investigation. Another plant-based therapeutic option with antioxidant and free radical-scavenging capability, *Rosa laevigata* (RLM), has been examined in a model of diabetic cataracts by Liu et al. using an immortalized LEC line (SRA01/04) [65]. RLM reduced ROS production and improved mitochondrial membrane potential via the stimulation of HO-1 expression and Nrf2 regulated gene in hyperglycemic SRA01/04 cells, suggesting that the protective effects of RLM are controlled by the PI3K/serine-threonine kinase (AKT) and Nrf2/ARE signaling pathways [65].

## 6. Epigenetics Modulation of Nrf2 Expression

Epigenetic factors that lead to protein misfolding and aggregation have been reported as contributors to cataract formation. Post-translational modifications of lens proteins cause protein destabilizations and subsequent aggregation [66,67]. Although cells have their mechanism of protection, environmental stress and mutant proteins can stimulate cataract formation. The rough ER is responsible for synthesizing the membrane, luminal or secretory proteins and then transporting it into the highly oxidized ER lumen. Stress can cause the misfolding of these proteins in the ER, leading to cataract development. The unfolded protein response intensifies crystallin and protein degradation and causes modification and aggregation in the downstream cascade [68]. As a mechanism of defense, ER-stressed cells increase their antioxidant ability to balance the ROS increase and homeostasis maintenance. Nrf2 is the crucial transcription factor that controls the genes that regulate the redox homeostasis [36].

The most common epigenetic modification induced by oxidative stress is DNA methylation, limiting the activity of promoters and enhancers genes in somatic cells during aging [69]. DNA methylation happens mainly at CpG dinucleotides. DNA methyltransferases transfer the methyl group to cytosine nucleotides, producing 5-methylcytosine, whose majority is found almost entirely within CpG dinucleotides located in mammalian somatic cell’s DNA [70]. There is not much information on the modifications in promoter DNA methylation patterns between normal lens aging and age-related cataracts [71]. The epigenetic modification represents a mechanism that selectively alters gene function as a response to conditions such as environmental and aging stresses. 

Gao et al. found DNA methylation of the *Keap1* promoter in non-cataractous human lens and cultured LECs, suggesting that Keap1 promoter demethylation is an age-dependent, crucial process for cataract formation [55]. Palsamy et al. described that methylated DNA sequence analysis of *Nrf2* and *Keap1* genes showed that the CpG dinucleotides in the *Keap1* gene are epigenetically modified but the same was not observed in *Nrf2* gene [71]. As mentioned previously, the Nrf2-Keap1 complex is a key cellular defender against oxidative stress, which is also connected with DNA hypomethylation in the *Keap1* gene in lens cataracts [36]. The loss of DNA methylation upregulates *Keap1* gene expression; a demethylated *Keap1* promoter leads to an increase in the expression of *Keap1* and enhanced levels of Keap1 protein [36]. Elevated levels of Keap1 stimulate Nrf2 degradation by ubiquitin-mediated proteasomal degradation and ER-associated degradation, leading to a decreased in Nrf2-dependent antioxidant defense and shifting the redox balance more towards lens oxidation [36,46,71,72,73,74]. Misfolded protein conformation then initiates misfolded crystallin aggregation production and, ultimately, cataract formation [36]. DNA hypomethylation in the *Keap1* promoter is close to 0% in the lens of individuals around 17 years of age but is up to 40% and 50% in the lens of individuals aged 60 and 75 years, respectively [36,71,73]. The loss of DNA methylation in aged populations (40–50%) is highly increased (90%) with cataractogenic stress in those who develop cataracts associated to age, suggesting that cataracts incidence is significantly enhanced with DNA hypomethylation [36,71,73].

## 7. Matrix Metalloproteinases Overexpression Induces Cataract Formation

In diabetic cataracts, a range of pathological changes of LECs directly impacts the disease [75]. Studies demonstrated that cataract LECs present a high expression of TGF-β1 linked with these epithelial cells’ differentiation and proliferation [76]. Additionally, modifications to LECs’ extracellular matrix formed by the lens capsule can influence cell differentiation and proliferation [18,77]. Matrix metalloproteinases (MMPs) belong to a family of enzymes that regulate tissue remodeling and are controlled by tissue inhibitors, known as tissue inhibitors of metalloproteinases (TIMPs), which regulate activity of enzymes and proenzymes activation. Constitutive expression of numerous MMPs and TIMPs has been observed in ocular tissues such as the lenses [78].

Matrix metalloproteinase 9, in particular, participates in the decomposition of LECs extracellular matrix and has been correlated with diabetic cataract [18,77]. Increased activation of MMP-2 and MMP-9 in lenses that suffered stress by oxidative processes, radiation, or TGF-β was observed in process of corneal wound healing and cataract development [14,15,16]. Evidence shows that MMP-2 and MMP-9 expression is stimulated in different cataract phenotypes, including subcapsular cataract (ASC) and posterior capsular opacification (PCO) [17,18]. Using MMP knockout mice, Korol et al. observed that MMP-9 knockout mice showed resistance to TGF-β-induced ASC formation, suggesting that inhibition of MMP activity through MMP inhibitors may help prevent some types of cataract, including ASC and PCO [79]. Studies demonstrated an increase in MMP-9 expression by stimulating the proteolytic cleavage of latent TGF-β and E-cadherin, leading to epithelial-mesenchymal transition (EMT), which is linked to lens opacity [80,81]. Moreover, MMP-9 activity levels in LECs were measured in patients with diverse age-related cataracts, showing that the main MMP-9 activity was found in cortical cataracts [82]. Numerous studies indicate that MMPs have a crucial function in cataract formation by the stimulation of intracellular β-crystallin aggregation and growth factor receptors shedding [83,84]. Thus, inhibitors of MMPs may have the potential to prevent and treat ASC, PCO, and cortical cataracts associated to age.

## 8. Association between Nrf2 Expression and MMP-9 Activity

Numerous proinflammatory cytokines (i.e., MMP-9) are overexpressed after NFκB activation by oxidative stress. This process of proinflammatory oxidative stress activates NFκB and causes overexpression of cytokines. To disrupt this cycle, the activation of the Nrf2/ARE system is crucial [85]. The link between the expression of Nrf2 and MMP-9 has been studied in different models. Mao et al. demonstrated that in Nrf2 function deficient mice, there was increased edema in the spinal cord, exacerbated inflammatory response, activation of NFκB, production of TNF-α, and expression of MMP-9 after the spinal cord injury, compared to the controls. The data suggested that Nrf2 expression may have a protective function by controlling inflammatory responses [86]. The Nrf2/HO-1 axis decreased the expression of two members of the MMP family; MMP-9 in macrophages and MMP-7 in epithelial cells of the human intestine, improving the disease state of inflammatory bowel disease [87,88]. In Nrf2-knockout mice with skin damage induced by UV-radiation, higher levels of MMP-9 were observed compared to the control, indicating that Nrf2 protects against UV radiation by reducing MMP-9 expression [89]. It was also reported that in inflammation and tumor cell invasion, NFκB signaling inhibition could reduce MMP-9 transcriptional activation [90]. Thus, during inflammatory processes, MMP production may be controlled directly by the Nrf2 pathway or by the effect of Nrf2 in the activation of the NFκB pathway [87].

## 9. Polyphenols and Nrf2 Activation

While the various polyphenols that have been discussed show potential in cataract treatment, there are currently no known studies connecting their antioxidant effects on Nrf2 activation concerning lens epithelia. However, a few studies have evaluated the Nrf2 activation capability of polyphenols in human retinal epithelial cells (RPE). Using cultured human ARPE-19 cells and primary RPE, Hanneken et al. found that quercetin protected RPE cells after oxidative stress exposure, and both quercetin and epigallocatechin gallate (EGCG) induced the nuclear protein expression of Nrf2 and HO-1 [91]. Sampath et al. investigated the cytoprotective effects of bioactive compounds isolated from ginger, apple, and tea, including EGCG, on methylglyoxal-induced carbonyl stress RPE. They showed that EGCG reduced the toxic effect of methylglyoxal [92]. EGCG was also reported to be a potent inhibitor of advanced glycation end products compared to the untreated group, and phloretin, an antioxidant chemical found in apples, significantly increased the translocation of Nrf2 to the nucleus and enhanced HO-1 expression compared to cells treated with MGO only [92]. Hu et al. examined thymoquinone (TQ) protective effect against H_2_O_2_-induced oxidative stress in RPE and demonstrated that TQ induced Nrf2/HO-1 signaling activation compared with the H_2_O_2_ induction group [93]. The results were further confirmed with si-Nrf2, where knockdown of Nrf2 abolished TQ’s protective effect (compared with TQ treatment group) on H2O2-induced oxidative damage suggesting that TQ protects RPE from oxidative stress via the Nrf2/HO-1 signaling pathway [93]. Studies such as these are needed in lens epithelia to substantiate further the ability of polyphenols to induce Nrf2, which, in turn, regulates the protective mechanism against cataract formation.

Another potential therapeutic plant-based compound that has been shown to involve the Nrf2 pathway is paeoniflorin (PF), a monoterpene glucoside compound extracted from *Paeonia lactiflora* roots [94,95,96]. Wankun et al. explored PF’s effects on oxidative stress induced by H_2_O_2_ and the mechanisms involved in Nrf2-related signaling pathways in human cultured ARPE-19 cells. MTT cell viability assay showed that PF effectively prevented H_2_O_2_-induced cell death in a dose-dependent manner and significantly inhibited H_2_O_2_-induced ROS production [96]. Although this study did not explore the direct effects of PF on NRf2, PF treatment was found to both significantly inhibit H_2_O_2_-induced caspase-3 activity and decrease phospho-p38 MAPK and phospho-ERK, suggesting that PF mediates its protective effects through Nrf2-related signaling pathways [96].

## 10. Polyphenols and MMP-9 Inhibition

Numerous studies have shown the inhibitory effect of polyphenols on the expression of MMP-9 [97] in different models. Using a rat model (middle cerebral artery occlusion), Tu et al. reported that baicalin, a flavonoid compound isolated from Chinese medicine radix of *Scutellaria baicalensis* Georgi, decreased MMP-9 expression, protected tight junction proteins, reduced blood brain barrier (BBB) damage, and attenuated brain edema [98]. In agreement, baicalin also attenuated the expression of MMP-9 in rats with intracerebral hemorrhage brains, decreased BBB damage, and brain edema [99]. It is not clear how baicalin reduces the expression of MMP-9, but it is known that inhibition of NFκB may reduce the transcription of the MMP-9 gene in stroke models, decreasing the expression of MMP-9 [99]. Additionally, MAPK signaling, more specifically, p38, may be involved in the diminished levels of MMP-9 [100]. Resveratrol, a natural phenol found in the Chinese herb *Polygonum cuspidatum* [101], reduced the expression of MMP-9 in rodent ischemic brains, decreasing edema and lessening BBB damage [102,103]. Using the molecular docking approach, resveratrol interacted with residues Glu 402, Ala 417, and Arg 424. It occupied the active site of MMP-9 [104], explaining the direct effect of the compound in a stroke model. Moreover, in vitro studies reported that resveratrol inhibited MMP-9 expression by activating peroxisome proliferator-activated receptor-α and inhibiting extracellular signal-regulated kinases [105,106]. Another polyphenolic compound extensively studied is curcumin, from the Chinese medicine Curcuma longa Linn [107]. Curcumin was reported to reduce the expression of MMP-9 in ischemic brains [108], possibly by the downregulation of NFκB activity, showing an indirect effect [109]. ’t Hart et al. demonstrated that apocynin, derived from the medical plant *Picrorhiza kurroa* [110], reduced BBB damage and protected tight junction proteins in a hyperglycemic rat (rat middle cerebral artery occlusion model) via downregulation of MMP-9/-2 [111,112]. Many other plant-derived compounds have been described to inhibit MMP-9 expressions, such as glycyrrhizin [113,114,115,116,117] and caffeic acid [118]. Mendonca et al. demonstrated that 1,2,3,4,6-Penta-O-galloyl-Beta-D-glucose, a polyphenolic compound found in many plants, inhibited proMMP-9 expression in LPS-activated BV-2 microglial cells. The MMP-9 expression may be associated with Alzheimer’s disease and the formation of senile plaques and neurofibrillary tangles, suggesting that MMP-9 could be a therapeutic target to treat brain inflammation [119]. These studies show evidence that polyphenols can modulate direct or indirectly the levels of MMP-9.

The effect of a citrus-fruit-derived flavonoid was also investigated in cataracts. Miyata et al. demonstrated that the intake of polymethoxylated flavones (PMFs) isolated from *Kaempferia. parviflora* caused a delay in cataract formation. These compounds inhibited the mRNA expression of MMP-9 stimulated by PMA, which is known to induce MMP-9 activity in LECs. Considering that MAPK signaling is a crucial mechanism to regulate the expression of MMP, the study showed that PMA increased MAPKs phosphorylation in LECs. At the same time, MAPK inhibitors, specifically for ERK1/2, p38, and JNK, inhibited MMP-9 expression and its subsequent activity in SRA01/04 cells [120]. The PMFs also inhibited phosphorylation of p38 and JNK in SRA01/04 cells, indicating that these flavones regulate MMP-9 mRNA expression via the PKC/p38 and PKC/JNK pathways in LECs [120]. Considering the crucial role of MMP-9 expression in cataract formation such as ASC and PCO, the authors suggested that the dietary intake of PMFs may have a therapeutic potential to prevent or help in the treatment of fibrotic cataracts.

## 11. Polyphenols and Cataract Formation

**Figure 2:**
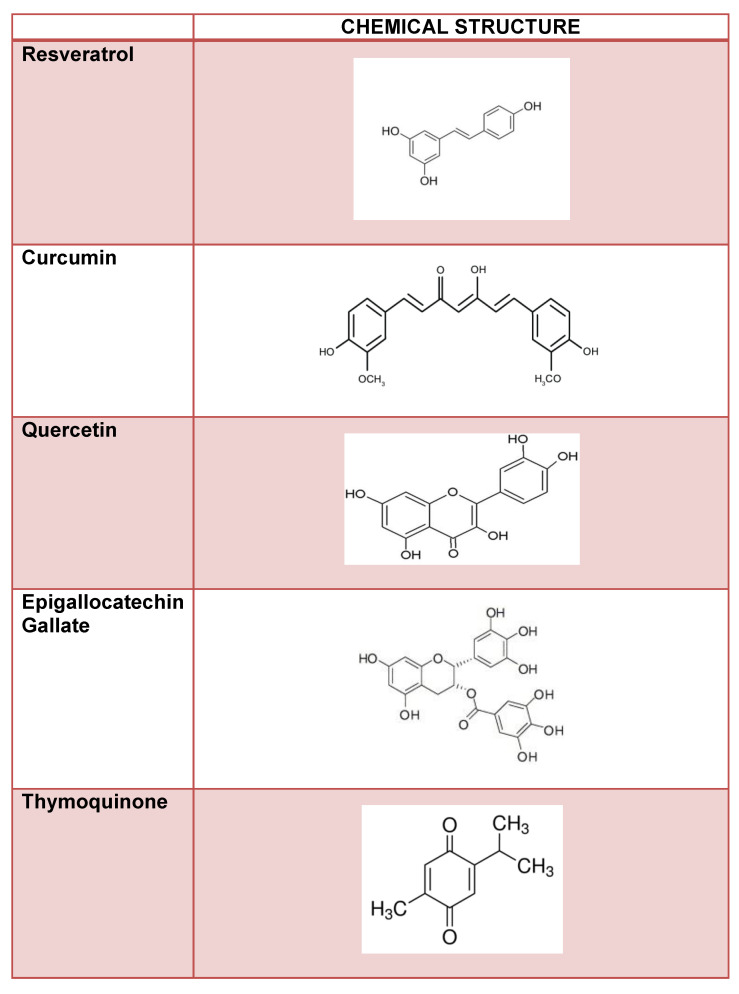
Flavonoid compounds and their chemical structure.

Non-enzymatic glycation is a mechanism associated to diabetic cataract development, and advanced glycation end-products accumulation with age may lead to lens opacity [4]. Thus, compounds with potent anti-glycating activity such as polyphenols are viable anti-cataract therapeutic options. Polyphenols are dietary antioxidants commonly found in foods such as fruits, vegetables, nuts, seeds, cereals, chocolate, and beverages such as tea, coffee, and wine [4]. With the growing interest in using food as medicine, polyphenol nutraceuticals have gained attention in treating cataracts. Caffeic acid, a naturally occurring cinnamic acid found in various plants such as coffee, pear, basil, oregano, and apple [4,121], has been shown to inhibit the formation of advanced glycation end-products [4,73,121]. Another naturally occurring cinnamic acid, ferulic acid, is found in vegetables, fruits, wheat, oats, and rice and has been shown to prevent advanced glycation end products [4]. Recent studies have investigated the therapeutic potential of several polyphenolic compounds on cataract development. The following describes these compounds (Figure 2).

### 11.1. Resveratrol

Resveratrol (trans-3,40,5-trihydroxystilbene) is a naturally occurring polyphenolic phytoalexin member of the stilbene family of compounds with two aromatic rings joined by a methylene bridge and is mainly found in seeds and skins of grape and fruit berries [122,123]. Its antioxidant activities include decreasing the production of ROS and increasing protection against oxidative stress [4,124]. Resveratrol has also been shown to prevent cataract formation by suppressing apoptosis of LECs [4,124]. To further examine the effects of resveratrol on diabetic cataracts in rats, Higashi et al. used a preclinical model of streptozotocin-induction of severe hyperglycemia to promote diabetic cataract formation in seven-week-old male Wistar rats. All lenses from the control group were clear throughout the experimental period [125]. Cataracts were observed in 77% of the lenses of diabetic rats two weeks after hyperglycemic-induction in the lens’s peripheral region [125]. In contrast, cataracts were detected in 75% of lenses of diabetic rats treated with 10 mg/kg/day resveratrol, and 60% of lenses of diabetic rats treated with 30 mg/kg/day resveratrol [125]. Cataract progressed with time throughout diabetic induction, with 40% of lenses developing hypermature cataracts nine weeks after induction; hypermature cataracts occurred in 17% of lenses in the 10 mg/kg/day resveratrol treatment group and 5% of lenses in the 30 mg/kg/day resveratrol treatment group. Although resveratrol did not entirely prevent diabetic cataracts’ appearance, it significantly delayed cataracts’ progression compared with controls [125]. Higashi et al. also examined the levels of sorbitol and protein carbonyls to measure polyol pathway activity and reactive oxygen-mediated protein oxidation [37,125,126,127]. Both sorbitol and protein carbonyl levels were increased in the lenses of diabetic rats compared to control rats [125]. Resveratrol blocked the increased protein carbonyl levels, but not of sorbitol, in diabetic lenses, suggesting that resveratrol delays diabetic cataracts’ progression in part by attenuating oxidative damage to lens proteins [125]. Although further work is needed to elucidate resveratrol’s detailed antioxidant mechanism, this work demonstrated that its anti-cataract effect appears to be partially due to decreased oxidative damage to lens proteins.

### 11.2. Curcumin

Derived from Curcuma longa L’s rhizome, curcumin has been used as an active ingredient of herbal remedies to treat various diseases with its antioxidant, anti-inflammatory antimutagenic, antimicrobial, and anticancer activity in traditional Chinese medicine and Ayurvedic medicine for thousands of years [128,129,130,131,132,133,134]. Turmeric is a well-known source of curcumin as a spice widely used in cooking. Despite its poor bioavailability, it is non-toxic and generally well tolerated at high doses of 8 to 12 g/day humans [128,135]. Concerning its antioxidant properties, curcumin acts through various mechanisms: it may scavenge ROS and reactive nitrogen species [128,136]; it may modulate the activity of enzymes responsible for the neutralization of free radicals such as glutathione (GSH), catalase (CAT), and superoxide dismutase (SOD) [128,137,138]; and it may inhibit enzymes that generate ROS, such as lipoxygenase/cyclooxygenase and xanthine hydrogenase/oxidase [128,137]. In cultured human LECs, Chhunchha et al. showed that curcumin inhibited peroxiredoxin 6, a pleiotropic oxidative stress-response protein [128,139]. Curcumin has been explored in numerous cataract models, demonstrating its ability to suppress oxidative stress induced by selenium and delay the formation of cataracts by inhibiting non-enzymatic antioxidant depletion in rat organ cultured lens [128,140]. Curcumin was also shown to delay diabetic cataract progression, significantly decreasing GSH levels and preventing the alteration of protein carbonyls, antioxidant enzymes such as glutathione peroxidase glucose-6-phosphate dehydrogenase (G6PD), thus preventing hyperglycemia-induced oxidative stress in rat lenses [128,141]. 

Recently, Cao et al. investigated the potential mechanism of the anti-cataract and cytoprotective effects of curcumin using sodium selenite-induced cataract in vivo and LEC in vitro models [142]. Methods included CCK-8 assay and flow cytometry to assess cell viability, cell apoptosis, and cell cycle in the in vitro studies along with RT-PCR and ELISA to analyze the expression of the following: heat shock protein 70 (HSP70), 8-hydroxy-2-deoxyguanosine (8-OHdG), catalase, malondialdehyde (MDA), SOD, and glutathione peroxidase (GSH-Px), caspase 3, Bcl-2 associated X (Bax), B-cell lymphoma 2 (Bcl-2), cyclooxygenase (Cox-2), c-met, and Slug [142]. In the in vivo studies, HSP70 levels and 8-OHdG and MDA activities were decreased in the lens from the curcumin treatment group compared with the control group [142]. Conversely, activities of CAT, SOD, and GSH-Px were significantly higher in the lens from the curcumin treatment group compared to the control group [142]. Cell viability and apoptosis were significantly increased, and caspase-3, Bax, and Cox-2 expression were decreased in LECs treated with curcumin compared to controls. These results suggest that curcumin attenuated selenite-induced cataract formation by reducing intracellular ROS production and protecting cells from oxidative damage [142].

### 11.3. Quercetin

Quercetin, which is also a flavonoid, is found in a diversity of fruits and vegetables and has been shown to protect against cataracts induced by H_2_O_2_ and retinal lesions induced by diabetes [122,143]. Quercetin-3-D-galactoside (hyperoside), a type of flavonoid generally found in *Hypericum perforatum* L., can inhibit oxidative stress by upregulating ERK activity in hydrogen peroxide (H_2_O2)-treated human LECs, which in turn increases Nrf2 expression and its antioxidant response [144]. Further studies on the anti-aging functions are needed to understand its full potential in protecting LECs against cataract formation. Park et al. recently examined the anti-cataract effect of eight dietary flavonoids, including quercetin, in a glycation-induced goat lens organ culture study [144]. The researchers analyzed lens transparency as the high refractive index; lens transparency is the essential prerequisite for visual acuity [144]. Quercetin, along with kaempferol and taxifolin, effectively maintained lens transparency and structural integrity of the glycation-induced cataractous lenses [144]. Together, this study’s results focus on the use of quercetin, kaempferol, and taxifolin as potential candidates for the management of glycation-induced cataract formation [144]. 

### 11.4. Epigallocatechin Gallate 

Epigallocatechin gallate (EGCG) represents more than 50% of the polyphenols that are found in green tea [122]. It has been shown to exhibit significant antioxidant properties through the inhibition of ROS-generating enzymes [122]. Chaudhary et al. performed a series of elegant structural and spectrophotometric analyses to assess the effects of EGCG on human γ crystallin aggregation in cataract formation. One study determined the effect of EGCG on H_2_O_2_-mediated oxidation of tryptophan (Trp) residues of a modified form of γ crystallin (HGCc) isolated from the human ocular lens cataracts. Oxidation of Trp is thought to be a key factor in HGC modification starting at the center of the lens, then spreading as oxygen diffuses to the center and is converted to H_2_O_2_, thereby damaging the lens proteins [145,146]. The fluorescence intensity of N-formyl kynurenine (NFK), one of the major oxidized products of Trp human crystallin [147], was used in this study to monitor the extent of Trp oxidation in the presence and absence of EGCG. HGCc showed significant emission at 339 nm due to Trp residues and a strong fluorescence at ~420 nm due to NFK when excited at 330 nm, confirming cataract presence due to Trp oxidation [145]. Using a fixed amount of 200 mM H_2_O_2_ to keep the ratio of protein and H_2_O_2_ at 1:100 and an excitation wavelength of 330 nm, a broad spectrum with a fluorescence maximum located at ~420 nm was observed in the absence of EGCG [145]. A gradual decrease in the fluorescence intensity of NFK was observed in the presence of increasing concentrations of EGCG from 0–16 mM, suggesting the EGCG could hinder H_2_O_2_-oxidation of Trp at concentrations well below the toxicity range of EGCG [145]. The researchers also studied the crystal structure to assess the docking of EGCG and demonstrated that EGCG is positioned between the two lobes of HGC in close proximity of Trp 157, Tyr 50, and Tyr 151 [145]. Thus, EGCG interacted with Trp of HGCc with high affinity accompanied by quenching of the Trp fluorescence [145]. In all, these studies showed that Trp oxidation is involved in oxidative stress-mediated cataract formation and the inhibitory potential of EGCG.

Another more recent study at Chaudhury et al. investigated the fibrillar aggregation of human γβ-crystallin in the absence and presence of EGCG using numerous techniques [148]. A previous study by this group showed that EGCG inhibited photooxidative damage of human γβ-crystallin [149]; thus, they continued to assess the inhibitory potential of ECGC on human γβ-crystallin aggregation. Human γβ-crystallin formed fibrillar aggregates at pH 2.0 (50 mMKCl/HCl) and were monitored in the absence and presence of EGCG [148]. Turbidity assays showed that native γβ-crystallin rapidly aggregated as depicted from the increase in the absorbance value at 350 nm of the protein in the absence of EGCG after 24h (~0.25 a.u.) and 48h (~0.6 a.u.). In the presence of EGCG, a noticeable decrease in the absorbance was seen (<0.1 a.u.) at 24h and 48h, suggesting that EGCG is capable of preventing aggregation [148]. Kinetic studies assessed γβ-crystallin aggregation at low pH and elevated temperature by measuring thioflavin T (ThT), a non-fluorescent dye in buffer/water whose fluorescence intensity is enhanced when it binds with a fibrillar cluster [150]. Native γβ-crystallin did not initially display any significant ThT fluorescence intensity at 485 nm (<175 a.u.). However, ThT fluorescence intensity increased significantly to >400% higher after 48h (>525 a.u.) [148]. In contrast, ThT fluorescence intensity was much lower in the presence of EGCG (<375 a.u.) at both 24h and 48h, indicating EGCG inhibited fibril formation. ANS fluorescence spectroscopy was also used to assess the tertiary structure of γβ-crystallin fibrils. There was no significant difference in ANS fluorescence intensity of native γβ-crystallin compared to γβ-crystallin fibril, which showed a strong ANS fluorescence and pronounced blue shift at 48h post-incubation [148]. A significant reduction of ANS fluorescence of γβ-crystallin incubated with EGCG (~175 a.u.) compared to native γβ-crystallin (~280 a.u.) was also noted [148]. These results show that hydrophobic patches on γβ-crystallin solvent are exposed under acidic conditions, resulting in conformational changes and partial loss of tertiary structure and suggesting that EGCG prevents the hydrophobic site exposure on the protein [148]. Circular dichroism spectroscopy further monitored the secondary structural changes of γβ-crystallin in the absence and presence of EGCG, with native human γβ-crystallin sample containing a mostly β-sheet secondary structure and a much smaller decrease in absorption minimum at ~218 nm in the presence of EGCG compared to no EGCG, suggesting that EGCG could protect the protein from being aggregated [148]. 

To visually confirm the fluorescence studies, electron microscopy studies were employed. Using high-resolution transmission electron microscopy (HRTEM), lower quantities of fibrils were seen in the presence of EGCG at 24 h compared to control, and the fibrils seemed to disintegrate into smaller aggregates after 48h [148]. Field emission scanning electron microscopy showed distinct, unbranched, long curly fibrils at lengths of ~1 mm at the onset of incubation in the absence of EGCG [148]. With fluorescence microscopy, γβ-crystallin showed amyloid fiber-like features of typical unbranched fibers of ~20μm diameter and few mm in length; no fibrillar features were detected in γβ-crystallin in the presence of EGCG [148]. These studies’ totality indicates that polyphenols such as EGCG prevents γβ-crystallin fibrillar aggregation under stressed environments in part by preventing strands from forming extended β-sheets [148]. Such approaches can help further identify the therapeutic properties of EGCG and gives information about how to design EGCG nutraceuticals to combat cataract formation. 

### 11.5. Nigella sativa and Thymoquinone

*Nigella sativa* oil (NSO), also known as black seed oil, belongs to the Ranunculaceae family and typically contains >30 w/w of fixed oil and 0.40–0.45 w/w of volatile oil; the volatile oil typically contains 18.4–24% thymoquinone (TQ) [151,152]. Both NSO and TQ have been reported to have strong antioxidant properties against oxidative damage induced by various free radical generating agents and have been used as nutraceuticals [151,152]. Two recent studies have evaluated the antioxidative effects of NSO and TQ on ionizing-induced cataract formation. Eye damage is widely observed in patients receiving total-body irradiation before bone marrow transplantation and ocular or head and neck cancers. A damaging, downstream effect of this type of radiation is ROS production, leading to cataract formation [151,153]. Both Demir et al. and Taysi et al. used similar approaches to investigate the antioxidant and radioprotective effects of NSO and TQ [151]. After the tenth day of total cranium radiation, both groups found the development of cataracts in 80% of the rats in the radiotherapy group [151,152]. Cataract rates dropped to 20% in NSO and 50% in TQ groups. They were limited to grade 1 and grade 2 based on Chylack’s cataract classification system [152,154], showing that NSO and TQ could prevent ionizing radiation-induced cataract formation [151,152]. Radiation-induced increases in xanthine oxidase [151], nitric oxide synthase, nitric oxide, and peroxynitrite [152] were prevented by NSO and TQ, suggesting that these substances could prevent irradiation-induced cataract formation by decreasing the lipid peroxidation, preserving antioxidant enzyme activities, and inhibiting free radical generation [151,152].

## 12. Conclusions

**Figure 3:**
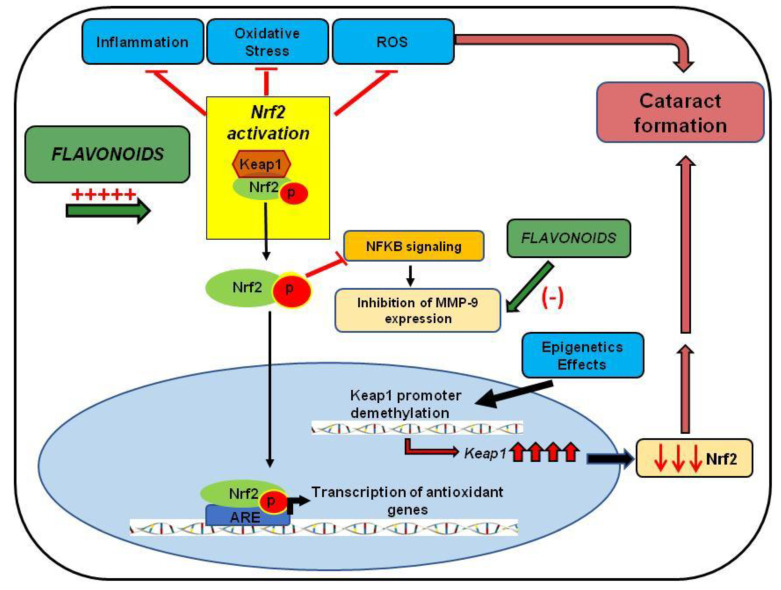
The protective effect of flavonoids in cataract formation. The diagram shows the effect of flavonoids on Nrf2 activation, leading to reduced inflammation, oxidative stress, and ROS, involved in cataract development. The figure also highlights Nrf2 modulation by epigenetic factors and the attenuation of MMP expression by flavonoids or by the inhibitory effect of the Nrf2 pathway in NFκB pathway activation.

In conclusion, these studies indicate that increased oxidative stress via inflammation, protein oxidation, unfolded protein response activation, DNA damage, and demethylation lead to injuries in the lens epithelia and, ultimately, cataract formation. The Nrf2/Keap1/ARE signaling pathway has emerged as one of the major cell defense mechanisms against oxidative stresses [35]. Chronic stressors suppress Nrf2-dependent antioxidant protection by overproduction of ROS and/or DNA damage and subsequent demethylation of Keap1, leading to loss of Nrf2 and ultimate cataract formation. As mentioned, natural compounds such as resveratrol, curcumin, phloretin, quercetin, ECGC, thymoquinone, and paeoniflorin have been shown to target directly or indirectly the Nrf2/Keap1/ARE signaling pathway (Figure 3). Thus, the development of Nrf2 inducers could have a profound impact on the treatment of cataracts.

The studies reviewed here have indicated the efficacy of Nrf2 activation and MMP-9 inhibition as critical therapeutic targets in treating cataracts. There are a sufficient number of studies describing the Nrf2 role as a key player in cellular defense mechanisms against aging and oxidative stress. Additionally, Nrf2 was reported as having a key role in inhibiting the overexpression of proinflammatory cytokines. It regulates the modulation of the NFκB pathway directly or indirectly, responsible for MMP expression (Figure 3). Moreover, this review discussed the regulatory effect of epigenetics in Nrf2 activation, indicating that DNA methylation changes may increase cataract incidence in the elderly (Figure 3).

Additionally, previous reports indicated that LECs are vulnerable not only to oxidative stress but also to apoptosis. As cited before, the imbalance between the ROS and antioxidants may exacerbate oxidative stress, changing the internal environment and leading to apoptosis of the lens. Both Nrf2 and MMP have been associated with cell apoptosis. Ma et al. using HO-1 inducers demonstrated that elevated levels of HO-1 stimulated the antioxidants activity and inhibited pro-apoptotic proteins, showing an indirect effect of Nrf2 in protecting the LECs against apoptosis [155]. In this regard, other studies showed that MMP might stimulate apoptosis by the disruption of a mitochondrial protein (connexin-43) and impairment of the membrane potential of the mitochondria [156]. Additionally, MMP seems to be stimulated by the diabetic environment, and it participates in several diabetic complications such as retinopathy. The activation of MMP-9, in particular, induced apoptosis in the retina capillary cells in the pathogenesis of diabetic retinopathy [157], showing that an elevation on MMP levels seems to be associated with the apoptosis process. Therefore, in agreement with this review, inducers of Nrf2 and inhibitors of MMP, such as the flavonoids, may have a beneficial effect against cataracts formation.

In conjunction with Nrf2 defense mechanisms, the research findings for the polyphenols reviewed here have demonstrated consistent beneficial outcomes concerning oxidative stress, inflammation, and epigenetic regulatory factors, crucial in developing cataracts. However, no studies show a direct correlation of the antioxidant, anticataract potential of polyphenols on Nrf2 activation and/or induction in lens epithelia. Thus, more work is needed to fully justify the superiority of Nrf2 as a therapeutic target specifically for lens cataract formation. Cataract surgical options may be limited by several reasons, including accessibility and affordability and surgery-associated complications. An important factor that may alleviate these barriers is supplementing the diet with flavonoids as an alternate treatment option for cataracts. This review provides evidence and strongly supports the use of flavonoid supplements that may increase Nrf2 activity and attenuate MMP-9 expression, which may be new targets to prevent or slow the lens’s cataract progression. However, more basic, and translational research is needed to understand these compounds’ effect on cataract development and progression.
